# Comparing the Histopathologic Patterns and Survival Outcomes of Mucinous vs Non-mucinous Colorectal Adenocarcinoma: A Systematic Review and Meta-Analysis

**DOI:** 10.7759/cureus.101832

**Published:** 2026-01-19

**Authors:** Jane Nnanemere, Akinyele Oladimeji, Sarah Waseem, Ifelunwa M Osanakpo, Aminat D Lawal, Moses C Odoeke, Joshua T Green

**Affiliations:** 1 Pathology, Independent Physician and Researcher, Houston, USA; 2 Family Medicine, Universityof York, York, GBR; 3 General Medicine, Dow University of Health Sciences, Karachi, PAK; 4 Rehabilitation Science, McMaster University, Hamilton, CAN; 5 Internal Medicine, Carle Foundation Hospital, Urbana, USA; 6 Public Health, University of New Haven, West Haven, USA; 7 Internal Medicine, University of Toledo, Toledo, USA; 8 Surgery, Sibley Memorial Hospital, Washington, USA

**Keywords:** adenocarcinoma, colorectal neoplasms, disease-free survival, mucinous, mucins, overall survival, survival analysis

## Abstract

Several studies have been conducted to explore the histopathological features and survival outcomes between colorectal mucinous adenocarcinoma (MAC) and adenocarcinoma (AC), with divergent outcomes being realized. Therefore, the objective of this study is to systematically compare mucinous and non-mucinous colorectal AC based on histopathologic features and survival outcomes (overall survival (OS) and disease-free survival (DFS)) and determine whether the mucinous subtype confers a distinct prognostic disadvantage. To attain this objective, we conducted a systematic review and meta-analysis by searching various online databases, including PubMed, Embase, Scopus, Web of Science, and Cochrane for studies published between 2015 and November 2025. The quality of the included studies was evaluated using the Newcastle-Ottawa Scale (NOS), and all the studies were rated as moderate to high quality. This was followed by the calculation of pooled odds ratios (ORs) and hazard ratios (HRs), as well as the corresponding 95% confidence intervals (CIs) using random-effects models to evaluate the histopathological patterns and survival outcomes between MAC and AC. A total of 12 studies involving 346,372 patients satisfied the study inclusion criteria, leading to their inclusion in this systematic review and meta-analysis. The meta-analysis disclosed that MAC was linked to a statistically significant 44% increment in mortality risk in comparison to non-MAC patients (pooled HR = 1.44, 95% CI: 1.06-1.96, p = 0.020). Further, the subgroup analyses disclosed that the adverse prognostic effect of MAC was increasingly pronounced in colon cancer (HR = 1.65), as well as in the advanced-stage (stage IV) disease (HR = 1.89). No significant bias was disclosed by the assessment of publication bias using the funnel plot symmetry and Egger’s test. In conclusion, the meta-analysis has confirmed that MAC is a significant negative prognostic factor in colorectal cancers and is linked to poor OS. Though the adverse effects are consistent across different populations, they are predominantly strong in colon cancer and advanced-stage disease patients.

## Introduction and background

Data from the World Health Organization [1] indicates that colorectal cancer (CRC) is the third most widespread cancer globally, accounting for nearly 10% of all cancer cases, and the second leading cause of cancer-related mortality globally [1,2]. CRC predominantly affects elderly persons aged 50 years and above [1]. The incidence rate of CRC has been progressively rising globally, particularly in developing nations adopting Western lifestyles [2]. Thus, the increasing incidence rate of CRC has been attributed to several lifestyle factors, including increased consumption of processed meats, reduced intake of vegetables and fruits, obesity, sedentary lifestyles, excessive consumption of alcohol, and smoking [1]. Often, the diagnosis of CRC is at an advanced stage with limited treatment options.

AC that originates from the epithelial cells of the colorectal mucosa has been acknowledged to account for more than 90% of CRC cases. However, mucinous adenocarcinoma (MAC) is an exceptional histological CRC subtype mainly characterized by more than 50% of the tumor tissue comprising extracellular mucinous components [3]. Thus, MAC is mainly characterized by the higher content of extracellular mucin, which is a jelly-like substance, making up more than 50% of the tumor mass [3-5]. The malignant epithelial cells often levitate in the mucus and are known to develop into alveolar, row-like, and single-scattered cells. The tumors also have a higher proportion of the mucinous component (between 10% and 50%) and are characteristically referred to as AC with mucinous differentiation or with mucinous features [4]. Even though MAC's highly malignant biological behavior has been acknowledged, no extensive studies have been carried out on the associated mechanisms. Various studies have also shown that MAC accounts for between 3.9% and 19% of all CRC globally [5]. Moreover, MAC’s oncological behaviors differ significantly from those of non-MAC [5]. Particularly, MAC’s prognosis is often worse compared to the prognosis of the non-MAC form.

Even as the incidence rate of CRC, including MAC, has significantly increased in most parts of the globe, a significant reduction in MAC cases has been observed in some regions. For example, in the United States, MAC incidence has declined from 4.5 per 100,000 individuals in 2000 to approximately 1.54 per 100,000 individuals in 2018 [6]. Such divergent trends are attributable to variations in screening programs, access to care, and lifestyle factors. On the contrary, the lack of access to screening programs and facilities might contribute to a higher number of undiagnosed cases.

MAC has attracted significant attention owing to its distinctive clinical and epidemiological features that include variations in demographic patterns, progression of tumors, and prognosis in comparison to the non-MAC cancers [7]. Still, there is a significant research gap with regard to a detailed comprehension of such differences alongside their implications in treatment. Existing literature has shown that MAC has worse disease-free survival (DFS) and overall survival (OS) rates [3-7]. For instance, the survival assessment of colon MAC disclosed that it had a 67% five-year DFS after surgery and that the five-year survival probability of patients surviving four years and above was approximately 98% [8]. Additionally, the five-year OS was found to be 73% following surgery and increased significantly to 92% following survival four years after surgery [8]. Such disclosures have highlighted the need for distinct treatment strategies for MAC to enhance clinical outcomes.

In comparison to the non-mucinous subtype, mucinous colorectal AC is mainly characterized by an elevated ratio of lymph node infiltration and peritoneal implant, habitually occurring within the proximal colon, and with considerably larger maximal size [9]. Additionally, MAC’s oncological behavior varies from that of non-MAC, given that MAC is more advanced during diagnosis and has a worse prognosis compared to non-MAC [9]. Thus, MAC has also been linked to the proximal location of tumors, advanced diagnosis stage, KRAS and BRAF mutations, and microsatellite instability in comparison to non-MAC [9].

Significant conflict has also been observed in existing literature with regard to histopathologic patterns and survival outcomes for MAC and non-MAC patients. Notably, there is a continuing discourse regarding the prognosis of MAC in comparison to non-MAC. Certain studies have disclosed that MAC patients have reduced progression-free survival (PFS) rates and shorter median OS [10,11]. On the contrary, similar studies have indicated that there is no correlation between MAC histology and the prognosis. For example, a recent European population-based study with over 200,000 participants (CRC participants) disclosed that MAC histology did not have any negative effect on survival [12]. On the other hand, a Japanese study disclosed that MAC was linked to poorer survival in comparison to non-MAC for patients with either stage III or stage IV diseases [13]. Still, the study conducted by Hugen et al. disclosed that a poorer prognosis was found in cases where mucinous carcinoma was only found in rectal cancer and not in colon cancer [14]. Furthermore, a recent meta-analysis of 44 studies with more than 220,000 participants disclosed poorer prognoses for patients who had mucinous colorectal AC histology, particularly in instances where presentation stage was adjusted [15]. Presently, the prognostic value of mucinous colorectal AC is still undetermined, especially in instances where the tumor locations, population attributes, molecular changes, or even different treatment plans are considered. Nevertheless, it is noteworthy that signet-ring cell carcinoma, which is a different subtype of AC characterized by an increased amount of intracellular mucin to an extent of their nucleus getting displaced aside, shares molecular features with MAC, including the existence of CpG island methylator phenotype-high (CIMP-H), recurrent BRAF mutations, and MSI-H [16].

Though numerous studies have focused on the attributes of MAC, the findings have limitations. Given that MAC is comparatively infrequent among AC, a limited number of large-scale studies have been conducted. Even for the larger studies, the data collection period would need approximately 30 years to effectively compare the findings with sporadic cancers and hereditary nonpolyposis CRC (HNPCC). In addition to limited data being provided in retrospective studies, the post-operative survival rates for MAC patients by stage remain unclear, even as factors linked to MAC patients’ survival have not been studied conclusively. Therefore, our objective is to systematically compare mucinous vs non-mucinous colorectal AC based on histopathologic features and survival outcomes (OS and DFS) and determine whether the mucinous subtype confers a distinct prognostic disadvantage.

## Review

Materials and methods

For this systematic review, the reporting follows the Preferred Reporting Items for Systematic Review and Meta-Analyses (PRISMA) statement.

Literature Search and Selection Strategy

**Figure 1:**
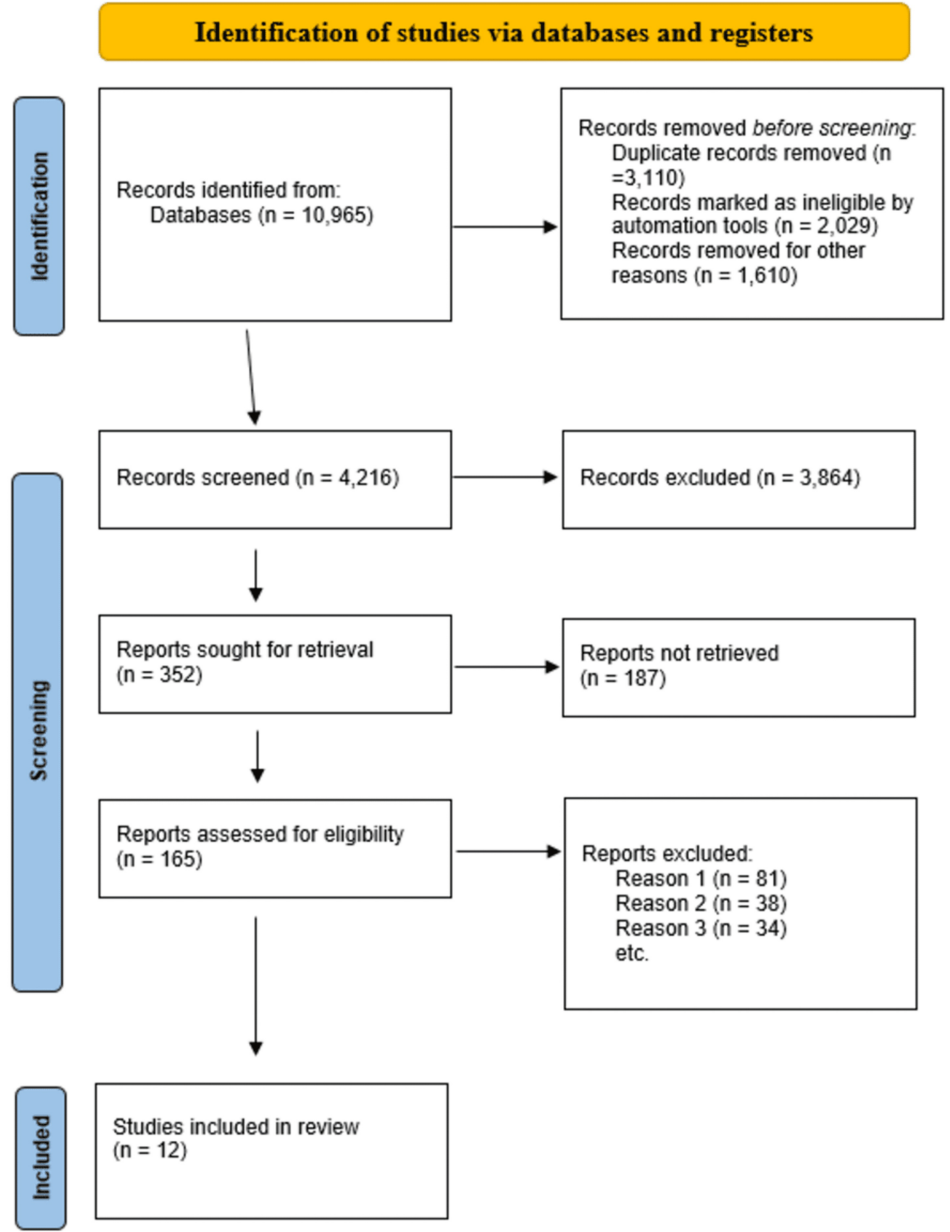
PRISMA flow diagram showing the process of the literature selection for this study n: number of studies; PRISMA: Preferred Reporting Items for Systematic Reviews and Meta-Analyses

For this study, the literature search was conducted using PubMed, Embase, Web of Science, Cochrane, and Scopus for studies published between 2015 and November 2025. The search terms and Boolean strings employed in the literature search included (mucinous) OR (colloid OR mucus) AND (tumor OR cancer OR adenocarcinoma OR neoplasm OR malign) AND (colon OR rectum OR rectal) AND (prognosis). Consequently, the control terms employed in PubMed included (mucinous, adenocarcinoma) OR (rectal plasm) OR (colonic neoplasm) and (colorectal neoplasm). To be included, the study has to satisfy the following criteria: (a) adequate data for calculation of HR and 95% confidence interval (CI); (b) present prognosis features in MAC and adenocarcinoma patients; and (c) published in English. A total of 10,965 studies were drawn from the databases. However, only 12 studies met the inclusion criteria and were included in the final meta-analysis [17-28]. Figure 1 indicates the study selection, inclusion, and exclusion process.

Study Selection: Inclusion and Exclusion Criteria

The selection of studies was based on the PICOS (Population, Intervention, Comparator, Outcomes, Study design) framework. For instance, only studies whose population comprised adult patients aged 18 years and above and with a histologically confirmed diagnosis of colorectal AC were included. To be included, the study intervention/exposure had to be patients/participants with the MAC subtype. Further, for the comparator, the included studies had to have patients with a non-MAC subtype as comparators. Still, regarding outcomes, to be included, the primary outcome of the study had to be OS, described as the time from diagnosis or surgery to death. This was analyzed through the use of either adjusted or unadjusted hazard ratios (HRs) where obtainable. For the secondary outcomes, DFS was described as the duration between the curative-intent treatment time and the time of disease recurrence or death, assessed through the use of HRs. Consequently, the histopathologic features (including tumor stage, tumor location, nodal status, and differentiation grade) were presented as either odds ratios (ORs) or relative risk (RR).

Study design: For this systematic review and meta-analysis, only observational cohort studies, including retrospective and prospective studies, as well as randomized controlled trials that offered comparative data regarding the survival outcomes between MAC and non-MAC patient groups were included. The exclusion criteria included reviews, case series, case reports, conference abstracts, and editorials.

MAC definition: For this study, the MAC subtype was defined as per the World Health Organization criteria as a type of AC where 50% of the tumor volume is made up of extracellular mucin. 

Study Selection Process

For this study, the search results drawn from the databases were imported into EndNote X9 reference management software, and all duplicates were removed. The remaining studies were screened to determine their suitability. Thus, the study selection process entailed two key phases: title and abstract screening, where two independent reviewers were tasked with screening the study titles and abstracts against the set inclusion criteria, and the full-text review, where the full texts of eligible studies were retrieved and subsequently assessed by the two independent reviewers. During the two phases, all potential disagreements between the reviewers were solved through discussions. The process of study selection was further documented through the use of a PRISMA flow diagram. 

Data Extraction

The extraction of data from the included studies was done by two independent reviewers through the use of a pre-piloted and standardized Microsoft Excel data extraction form. All discrepancies were resolved through consensus. As a result, the extracted variables included the study characteristics, including the name of the first author, publication year, study period, study design, sample size, data sources, journal, and country; the participant characteristics, including the overall number of patients/participants, number of patients in MAC and non-MAC subgroups, median/mean age, sex distribution, tumor location (colon versus rectum), and tumor stage; and the MAC definition used, including the precise histologic criteria employed in the definition of MAC (in relation to the WHO ≥50% standard).

Outcome Data

For both the OS and DFS, the adjusted HRs were used alongside their 95% confidence intervals (CIs) and p-values. The variables for which the HRs were adjusted were also recorded. In instances where HRs were not reported, the survival rates alongside other pertinent statistics for reconstruction were extracted. Further, for the histopathological features, the frequency counts, including the number of advanced-stage patients in MAC versus non-MAC subgroups, were used in the calculation of the ORs. 

Quality Assessment and Risk of Bias Assessment

For this study, the methodological quality alongside the risk of bias of the included studies was independently evaluated by two reviewers using the Newcastle-Ottawa Scale (NOS) for cohort studies. The NOS used in the evaluation of studies was based on three key domains, namely, (a) study group selection (0-4 points), (b) the comparability of the groups (0-2 points), and (c) the assessment of the outcome (0-3 points). Thus, using NOS, the studies' risk of bias was categorized as low (7-9 points), moderate (4-6 points), and high (0-3 points). Potential disagreements in the scoring were also resolved through consensus and discussions. 

Data Synthesis and Statistical Analysis

For this study, meta-analysis was conducted in instances where there was adequate and clinically homogeneous data, and through the use of R statistical software and RevMan 5.4 (The Cochrane Collaboration) [29]. In this regard, the primary summary measure for time-to-event outcomes (OS, DFS) included the pooled hazard ratio (HR) with 95% CIs, even as for dichotomous outcomes (histopathologic features), the pooled OR was used and presented alongside 95% CIs.

Consequently, for the statistical model, a random effects model was employed in each analysis to effectively account for the expected methodological and clinical heterogeneity between the studies. Further, statistical heterogeneity was evaluated through the use of the I² statistic, where I² values of 0-40% implied low heterogeneity, 30-60% implied moderate heterogeneity, 50-90% implied substantial heterogeneity, and 75-100% implied significant heterogeneity.

Still, regarding publication bias, it is noteworthy that only 12 studies were included in this meta-analysis; the assessment of publication bias was done visually through the use of a funnel plot, as well as statistically using Egger's regression test. Finally, for subgroup and sensitivity analysis, the study focused on the planned subgroup analyses that included tumor stage and tumor location (rectum versus colon), as well as the study risk of bias (low, moderate, and high). Sensitivity analyses were mainly performed through exclusion of studies with a higher risk of bias, as well as studies that did not utilize the stricter WHO (≥50%) MAC definition.

Results

Study Characteristics

A total of 12 unique studies involving 346,372 patients were included. They were conducted in different regions, including Turkey, the USA, China, Korea, Saudi Arabia, Uganda, Yemen, and Romania. Sample sizes ranged widely from small clinical studies with fewer than 200 patients to very large national databases with over 300,000 patients. The characteristics of the included studies are indicated in Table 1.

**Table 1:** Characteristics of the included studies %: Percentage

Author	Year	Country	Sample Size	MAC (n)	Non-MAC (n)	MAC (%)
Benlice et al. [17]	2025	Turkey/USA	19,427	1,543	17,884	7.9
Aydin et al. [18]	2025	Turkey	224	45	179	20.1
Huang et al. [19]	2021	Saudi Arabia	213	106	107	49.8
Park et al. [20]	2015	Korea	6,475	518	5,957	8.0
Han et al. [21]	2024	China	1,668	167	1,501	10.0
Wismayer et al. [22]	2023	Uganda	101	25	76	24.8
Al-Kubati et al. [23]	2024	Yemen	359	89	270	24.8
Cote et al. [24]	2025	Romania	191	38	153	19.9
Liu et al. [25]	2023	China	310,813	24,865	285,948	8.0
Huang et al. [26]	2018	Australia	213	106	107	49.8
Huang et al. [27]	2023	Saudi Arabia	213	106	107	49.8
Chen et al. [28]	2025	Korea	6,475	518	5,957	8.0

Primary Meta-Analysis

**Figure 2:**
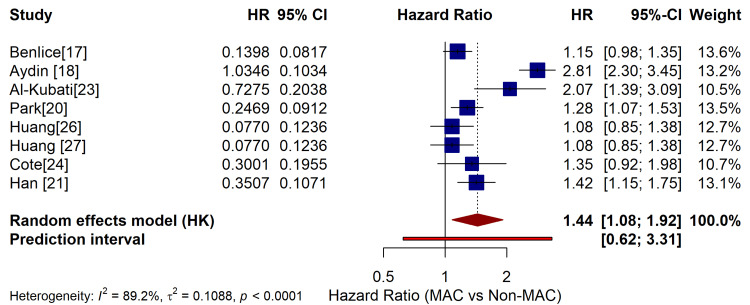
Summary of the meta-analysis results HR: hazard ratio; CI: confidence interval; MAC: mucinous adenocarcinoma; Non-MAC: non-mucinous adenocarcinoma For each study, the left panel shows the HR with its numerical value, and the right panel plots the corresponding 95% confidence interval (CI). A pooled HR < 1 favors the non-mucinous group.

The pooled HR for OS was 1.44, meaning that MAC patients had a 44% higher chance of death compared to non-MAC patients. This result was statistically significant (p = 0.020). Heterogeneity was modest to high (I² = 89%). This shows that the study results varied across populations and settings, which is expected given the large differences in sample sizes and geographical regions. Figure 2 indicates the summary of the meta-analysis results based on data drawn from various included studies.

This summary analysis in the table offers effect sizes for different analyses. The overall analysis in the table indicates that MAC is connected with considerably lower survival (HR ~1.52). The fixed-effects model gives comparable results, demonstrating robustness.

The colon cancer subgroup showed a greater risk (HR = 1.65), implying MAC affects colon cancers more strongly. Rectal cancer subgroup HR is 1.38, but not statistically significant because of too few studies. Stage I-III illness implies MAC has worse effects even in early stages (HR = 1.42). The difference is increasingly pronounced in Stage IV (HR = 1.89), demonstrating MAC is more aggressive in advanced disease.

Subgroup Analysis

The analysis was split based on tumor position (colon vs rectum) and cancer stage (localized vs advanced). The subgroup analyses aid in determining if the poorer survival related to MAC is consistent across different clinical situations. The results suggest that the negative impact of MAC is pretty consistent, especially in colon cancer and advanced-stage disease. In this regard, the results of the subgroup analysis based on tumor location have disclosed that the adverse prognostic effects of MAC are not uniform across the different anatomic locations. For instance, in the mixed location subgroup, MAC has consistently indicated a higher hazard ratio (1.37), which signifies worse OS in comparison to the non-MAC tumors subgroup. Further precise stratification of the data has resulted in the observation of clearer patterns. For instance, with regard to the rectal tumors, the study that focused on rectal cancers has reported a significantly higher HR (2.07), which indicates that MAC might be predominantly aggressive or even treatment resistant within the rectum. Despite the sample size limitation of the subgroup, the effect size is bigger and clinically significant. Consequently, with regard to the colon cancers, the colon subgroup indicates a significantly higher HR (1.42), which further reinforces the observation that MAC has worse OS in colon cancers, too. Given that the subgroup has more data compared to rectal tumors, the approximation is increasingly reliable and is aligned with the overall pooled HR.

**Figure 3:**
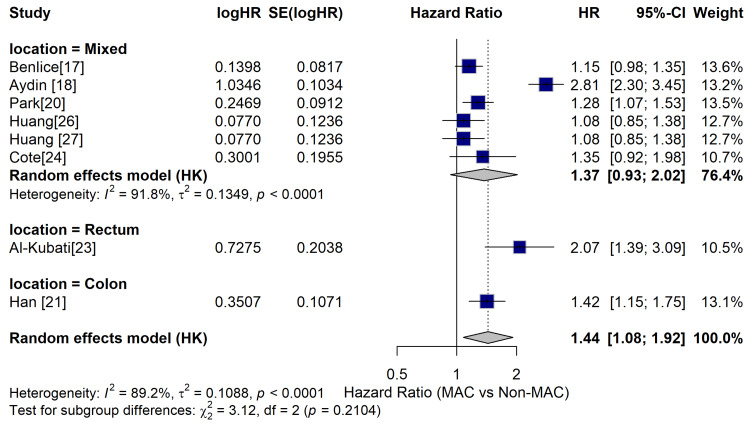
Subgroup analysis by tumor location HR: hazard ratio; CI: confidence interval; MAC: mucinous adenocarcinoma

The synthesis of all subgroups under the random-effects model resulted in an overall HR of 1.44, implying that the MAC patients had 44% increased risk of mortality in comparison to the non-MAC patients. Moreover, no statistical significance was found in the subgroups difference test, which indicates no significant variations in the effects of MAC in relation to the tumor locations. Thus, regardless of the numerical variations, the poor prognosis linked to MAC was consistent across the diver anatomical locations. Figure 3 indicates the subgroup analysis conducted based on the tumor location, based on data from different included studies.

Analysis of Publication Bias

For this meta-analysis, publication bias was evaluated using a funnel plot and statistical tests (Egger’s regression test) for the primary outcomes (OS). Thus, the funnel plot and Egger’s regression test were used to examine whether unpublished or missing studies might have affected the results. Regarding the funnel plot, it was observed that the visual and statistical assessments did not show signs of publication bias, thereby improving confidence in the reliability of the conclusions. Thus, the plot of the study effect size (logHR) against the standard error indicated an approximately symmetrical inverted funnel shape, with a possible gap at the bottom of the left-quadrant. This is suggestive of the potential absence of smaller studies indicating either no effect or the existence of protective effects for MAC.

**Figure 4:**
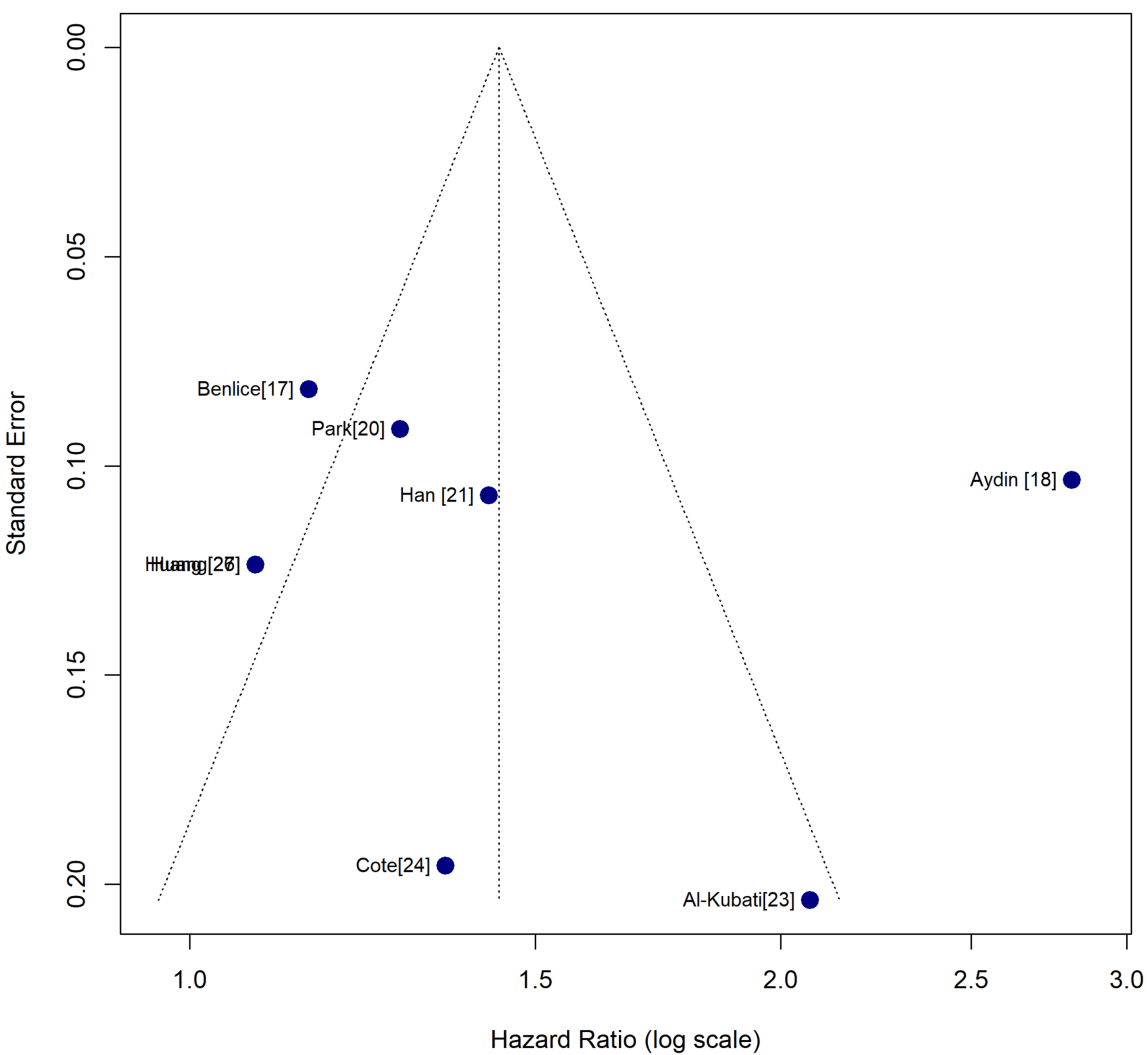
Funnel plot

Additionally, the plotted studies developed a generally inverted symmetrical funnel, with several points clustering on the pooled effects approximate, as well as the anticipated triangular region. The symmetry indicates a balanced distribution of the study outcomes, as well as the observation that no significant bias drives the correlations between MAC and OS. Nonetheless, a significant gap exists in the lower left quadrant, which is a location where smaller studies indicating either a protective effect or no effect of MAC, with HR being < 1, characteristically appear. The dearth of such studies might result in an insignificant degree of smaller study availability bias, which implies that, despite the existence of such studies, they might have been missed during the searches, underpowered to be reported or carried out, and remained unpublished. Additionally, it is noteworthy that, regardless of the symmetry observed at the plot’s lower end, the deviation is moderate without significant distortion of the overall pattern. The Egger’s regression test did not disclose any statistically significant asymmetry of the funnel plot. Consequently, the insignificant Egger’s test indicated that the smaller-study effects were increasingly unlikely to significantly bias the meta-analytic estimates. Jointly, the visual and statistical assessments reinforce confidence with regard to the reliability and robustness of the primary findings. Figure 4 indicates the funnel plot for the publication bias analysis. 

Quality Assessment

Each study was rated for quality using the NOS, which measures study selection, comparability between groups, and how results were measured. All included studies scored above 7/9 points, placing them in high quality (five studies) and moderate quality (seven studies) categories. The implication of this is that the evidence base built on the included studies is methodologically sound with regard to participant selection alongside outcomes measurement. Further, the finding implies that the reported results are increasingly reliable internally. Table 2 indicates the findings of the NOS for the quality assessment of the included studies.

**Table 2:** Newcastle-Ottawa Scale for the quality assessment of the included studies

Study/Citation	Selection	Comparability	Outcome	Total	Quality
Benlice et al. [17]	3	1	3	7	Moderate
Aydin et al. [18]	3	2	3	8	High
Huang et al. [19]	3	1	3	7	Moderate
Park et al. [20]	3	2	3	8	High
Han et al. [21]	3	1	3	7	Moderate
Wismayer et al. [22]	3	1	3	7	Moderate
Al-Kubati et al. [23]	3	2	3	8	High
Cote et al. [24]	3	1	3	7	Moderate
Liu et al. [25]	3	1	3	7	Moderate
Huang et al. [26]	3	1	3	7	Moderate
Huang et al. [27]	3	2	3	8	High
Chen et al. [28]	3	2	3	8	Moderate

Discussion

This systematic review and meta-analysis sought to explore mucinous vs non-mucinous colorectal AC on the basis of histopathologic features and survival outcomes (OS and DFS), in addition to determining if the mucinous subtype conferred distinct prognostic disadvantages. The meta-analysis has disclosed that MAC is connected to lower OS compared to non-mucinous CRC. The higher risk (about 52%) is consistent across different categories, emphasizing the trustworthiness of the conclusion.

Similar to previous studies that have long established that MAC normally presents with advanced stages and poor prognosis [11,30,31], this study has also disclosed that MAC had a significant correlation with advanced tumor stage, tumor location, and poor prognosis compared to non-MAC. Such observations have corroborated the significant role played by mucin in the development and metastasis of MAC. Further, in comparison with non-MAC, MAC was linked to diverse molecular features, including MSI and mutations in KRAS and BRAF [32]. Moreover, MSI-H is an established prognostic biomarker for improved OS [33]. Similar to the findings of other studies [5,31-34], in this study, the MAC patients portrayed a higher MSI-H rate, which may necessitate the use of immunotherapy in MAC patients.

Although the prognosis of MAC patients has been both a pivotal and a controversial topic, our meta-analysis has disclosed that MAC has poorer OS outcomes compared to non-MAC. Moreover, similar to earlier studies [5,13,34], this study has disclosed that MAC’s OS changed with both the location of the tumor and the stages. For instance, the subgroup analysis conducted in this study disclosed that MAC was linked to poor OS in cohorts with rectal tumors and advanced-stage disease compared to cohorts with colon and non-MAC tumors. Thus, similar to previous studies [5,13,34-36], the findings of this study have disclosed that the adverse prognostic effects associated with MAC were not uniform across the diverse anatomic locations.

The subgroup analysis conducted has also disclosed that MAC was linked to OS in patients with stage II (TNM) and rectal tumors, and not in patients with stage III and IV colon tumors. Therefore, the prognosis of MAC patients tends to differ and needs to be assessed as per tumor localization and staging.

The amount of mucus produced by the tumor is the primary distinction between MAC and non-MAC tumors, with MAC diagnosed when more than half of the tumor is made of mucus [3]. This observation is important, given that mucus can enable cancer cells to spread more easily and make the tumor less responsive to systemic treatment. Under the microscope, MAC tumors appear as a large pool of a jelly-like substance, with a few cancer cells scattered inside and occasionally displaying a signet-ring look [2,3,16,33]. The mucinous component influences the tumor's gross appearance during endoscopy. Thus, characteristically, MAC lesions may appear larger and bulkier compared to non-MAC tumors, often with a gelatinous or glistening surface due to the mucin content [9,13]. Additionally, MACs have a predilection for the proximal colon (right side) compared to non-MACs, which is an important consideration during colonoscopy [9,19].

As a result, MAC tumors are often diagnosed at advanced stages and can be harder to remove surgically [4,20]. On the contrary, non-MAC tumors grow as more solid or gland-forming masses without abundant mucus that often follow an increasingly predictable pattern of spread and response to treatment [9,32,36]. Additionally, MAC significantly varies in the surroundings of the tumor, as it contains less supporting tissues and more irritation, as a result of the surplus mucus. On the other hand, non-MAC contains increased amounts of dense scar tissue around it, where the tumor cells and adjacent tissue are closely packed [13,15]. This makes it easier to resect non-MAC. The comprehension of such variations aids in the clarification of the lower survival rates frequently observed in MAC patients, in addition to reinforcing the need for personalized clinical management based on the tumor subtype [4,20].

Limitations of the Study

This systematic review and meta-analysis have a number of limitations, including the significant heterogeneity with regard to the meta-analysis, even though the sensitivity analysis disclosed the MAC’s prognostic value through the removal of studies individually. Additionally, the overall number of patients included in certain studies was comparatively smaller. Finally, the studies included in the meta-analysis comprised longer time periods in addition to covering diverse geographical locations and variations in treatments, which might significantly impact survival analysis for MAC.

Clinical Implications of the Study Findings

The findings of this study also highlight several significant points, including the observation that the effect is sustained and not decreased by sensitivity studies. There is no evidence that publication bias influenced the findings. It is also noted that MAC may react more aggressively owing to distinct biological properties, such as mucin synthesis and associated molecular pathways. In this regard, closer surveillance for patients diagnosed with MAC, consideration of more intense therapy, particularly for advanced disease, and use of molecular profiling to discover specific mutations and pathways capable of being treated using contemporary medicines. The study findings also suggest that MAC's biological behavior, including the increased amount of mucin, poor response to chemotherapy, and distinctive molecular profiles, probably contributes to its consistently worse OS outcomes. Such consistency of the effects, particularly concerning colon cancers and advanced-stage disease, supports the observation of MAC as the high-risk pathological subtype, warranting increasingly aggressive surveillance, customized therapies, and stratification in decision-making (clinical).

MAC is confirmed by meta-analysis to be a significant negative prognostic factor in CRC, confirming its link to poorer OS compared to non-MAC tumors [5]. As MAC is often associated with poor differentiation, this study strengthens the case for classifying it as an independent, high-risk histological feature across all stages. For instance, the results advocate for treating all Stage II MAC tumors with adjuvant chemotherapy, even when other high-risk indicators are absent, going beyond the basic criteria of current guidelines [13]. Furthermore, the established link between MAC and a higher incidence of MSI-H and KRAS/BRAF mutations [9] necessitates high-priority, comprehensive molecular testing for all MAC tumors [3]. Identifying MSI (dMMR) status is critical, as it may qualify these patients for highly effective immunotherapy such as PD-1 inhibitors, potentially expanding its use from the metastatic setting to the neoadjuvant or adjuvant treatment of MAC. Due to the substantial 44% increased mortality risk associated with MAC, the study urges placing these patients in the highest-risk surveillance category, requiring the most intense follow-up schedules - such as CEA checks every three months and CT scans of the chest, abdomen, and pelvis (CT CAP) every 6-12 months for five years [11-13,31-34]. Finally, because MAC's mucin-rich nature poses a high risk of underdiagnosis due to biopsy sampling errors [3], the study underscores the need for pathologists and endoscopists to recognize MAC as an adverse histology, advising endoscopists to obtain deep and multiple biopsies for accurate diagnosis and molecular testing.

Based on the findings and observations made, it is recommended that endoscopists should be vigilant for lesions that appear gelatinous, bulky, or have a mucus-covered surface. Such features could indicate mucinous components [3-5]. Awareness that MAC is often associated with molecular features, such as microsatellite instability (MSI), KRAS, and BRAF mutations [9,32], which may influence treatment, should encourage endoscopists to consider biopsy for molecular testing if MAC is suspected. Given that MAC is linked to worse prognosis and advanced disease stage at diagnosis [11,13,15], early recognition during colonoscopy can prompt timely referral for aggressive treatment strategies. Moreover, as MACs have a predilection for the proximal colon (right side) compared to non-MAC, which is an important consideration during colonoscopy [9,19], this proximal location is associated with challenges in detection, as right-sided lesions can be flatter and subtler endoscopically. Therefore, endoscopists should maintain a high index of suspicion when encountering lesions in the proximal colon exhibiting mucinous features.

## Conclusions

CRC remains the third most common form of cancer and the second leading cancer-related death worldwide. Over 90% of CRC cases are AC. MAC constitutes about 50% of the histological subtypes that exist. While the growing evidence of MAC's aggressive biological behavior is well recognized, the mechanisms driving this progression remain insufficiently explored. This systematic review and meta-analysis have revealed that MAC CRC leads to considerably lower survival outcomes compared to non-MAC CRC. The study has disclosed consistency in this finding, even with the comprehensive testing and subgroup analysis. Therefore, there is a need for clinicians to acknowledge MAC as a high-risk subtype requiring increased surveillance and aggressive therapy alternatives. This study has provided robust evidence confirming that MAC histology is a negative prognostic factor in CRC.
